# Racial Disparities and Outcomes of Percutaneous Coronary Interventions in Patients Above 65 Years in America: A Systematic Review

**DOI:** 10.7759/cureus.42457

**Published:** 2023-07-25

**Authors:** Olawale O Olanisa, Panah Parab, Priti Chaudhary, Sonia Mukhtar, Ali Moradi, Athri Kodali, Chiugo Okoye, Dhadon Klein, Iman Mohamoud, Lubna Mohammed

**Affiliations:** 1 Internal Medicine, California Institute of Behavioral Neurosciences and Psychology, Fairfield, USA; 2 Internal Medicine, Lahore Medical and Dental College, Lahore, PAK

**Keywords:** bare-metal stents (bms), percutaneous coronary intervention (pci), drug-eluting stents (des), coronary artery disease (cad), end-stage renal disease (esrd), door-to-balloon time, st-segment elevation myocardial infarction (stemi), acute mi

## Abstract

This systematic review aims to examine the racial disparities and outcomes of percutaneous coronary interventions (PCIs) in patients above 65 years in America. The review follows the Preferred Reporting Items for Systematic Reviews and Meta-Analysis (PRISMA) guidelines 2020 and includes a comprehensive search strategy, study selection, data extraction, and quality assessment. The search strategy identified 10 relevant articles that were included in the review. The findings indicate that racial disparities exist in access to PCI, door-to-balloon (DTB) time, procedure utilization, and outcomes among elderly patients. African American and Hispanic patients were found to experience longer door-to-balloon time and lower rates of PCI utilization compared to White patients. Moreover, racial and ethnic minorities had worse clinical outcomes, including higher mortality rates and increased risk of major adverse cardiovascular events. The review also highlights the impact of Medicaid expansion on reducing disparities in access, treatment, and outcomes for patients with acute myocardial infarction (AMI). However, limitations in data availability and representation of racial and ethnic minorities in clinical trials were identified. The discussion section provides a robust analysis of the findings, exploring potential underlying factors contributing to the observed disparities. The review concludes that addressing racial disparities in PCI outcomes among elderly patients is crucial for achieving equitable healthcare delivery and improving cardiovascular health outcomes in America.

## Introduction and background

Racial disparities in healthcare, particularly in cardiovascular disease (CVD), have been a topic of concern and investigation. Cardiovascular diseases, including coronary artery disease (CAD), are a leading cause of morbidity and mortality among older adults in the United States. Percutaneous coronary interventions (PCIs), such as stenting and angioplasty, have revolutionized coronary artery disease management and significantly improved patient outcomes. However, growing evidence suggests that racial disparities exist in the utilization of PCI and subsequent developments, particularly among patients above 65 years of age [1]. Understanding and addressing these disparities is crucial to achieving equitable healthcare and optimizing patient outcomes. This systematic review examines the existing literature on racial disparities and outcomes of PCI in patients above 65 years in America [2].

These disparities result from factors such as socioeconomic status, cultural beliefs, healthcare system barriers, and implicit biases, leading to significant differences in access, treatment, and outcomes among racial and ethnic groups [3]. Investigating potential racial disparities in the utilization and outcomes of PCI among older patients becomes imperative to improve healthcare equity. Concrete data reveals that racial minorities often face higher rates of chronic conditions, leading to unequal health outcomes. For instance, Centers for Disease Control and Prevention (CDC) data showcases higher diabetes prevalence in African American and Hispanic populations. Studies have indicated that older racial minority patients may encounter barriers in accessing care, resulting in delays in diagnosis and treatment. Notably, research published in the New England Journal of Medicine has shown that African American patients with coronary artery disease were less likely to receive timely interventions such as PCI compared to White patients, potentially leading to worsened health conditions and increased mortality rates [4]. Black adults face higher burdens of cardiovascular risk factors, being over twice as likely to die of CVD compared to Whites, while American Indians are 1.5 times as likely to be diagnosed with coronary heart disease as Whites. Structural racism is cited as the root cause of these disparities. Addressing these issues through research and interventions is crucial to improve healthcare access and outcomes for all. Cardiovascular diseases claim over 650,000 lives annually in the USA, straining the healthcare system and the economy, highlighting the urgency of promoting a more just and equitable healthcare system for all [5]. Understanding the complex interplay of these factors is essential for developing targeted interventions to reduce disparities and improve healthcare equity. The elderly population is particularly vulnerable to cardiovascular diseases, emphasizing the necessity of investigating whether racial disparities exist in the utilization and outcomes of PCI among older patients. By identifying these disparities, we can develop strategies to enhance access, optimize treatment, and improve outcomes for all patients, regardless of their race or ethnicity [6,7].

This systematic review will follow the Preferred Reporting Items for Systematic Reviews and Meta-Analysis (PRISMA) guidelines, ensuring a rigorous and transparent approach to data selection, extraction, and synthesis. The selected articles will be critically evaluated to assess the quality of evidence, study strengths, and limitations. A comprehensive search strategy has been employed to capture relevant studies published up until the knowledge cutoff date of September 2021. The findings of this systematic review will contribute to the existing literature by summarizing the current evidence on racial disparities and outcomes of PCI in patients above 65 years in America. By identifying gaps in knowledge and understanding, this review will inform future research and policy efforts aimed at reducing disparities and achieving equitable healthcare for older patients with coronary artery disease. Ultimately, the goal is to improve patient outcomes and promote healthcare equity among diverse populations.

## Review

Methods

Search Strategy

This systematic review followed the Preferred Reporting Items for Systematic Reviews and Meta-Analysis (PRISMA) 2020 guidelines. A comprehensive search strategy was developed to identify relevant studies. The following databases were searched: PubMed, Embase, Research Gate, MEDLINE, ScienceDirect, Scopus, and Google Scholar. The search terms included keywords related to racial disparities, percutaneous coronary interventions, outcomes, and elderly patients. The search strategy was conducted using Boolean operators and Medical Subject Headings (MeSH) terms. The search strategy resulted in a total of 231 potential articles. After applying the inclusion and exclusion criteria, a total of 10 articles were selected for the systematic review.

Inclusion and Exclusion Criteria

For the study selection criteria, the following inclusion criteria were utilized. Firstly, studies conducted in the United States were considered. Secondly, the focus of these studies needed to be on racial disparities in outcomes of percutaneous coronary intervention (PCI) procedures. Additionally, studies involving patients aged 65 and above were included. Furthermore, only articles published in English were included in the selection process. Lastly, original research articles were considered, while reviews, commentaries, and editorials were excluded.

**Figure 1:**
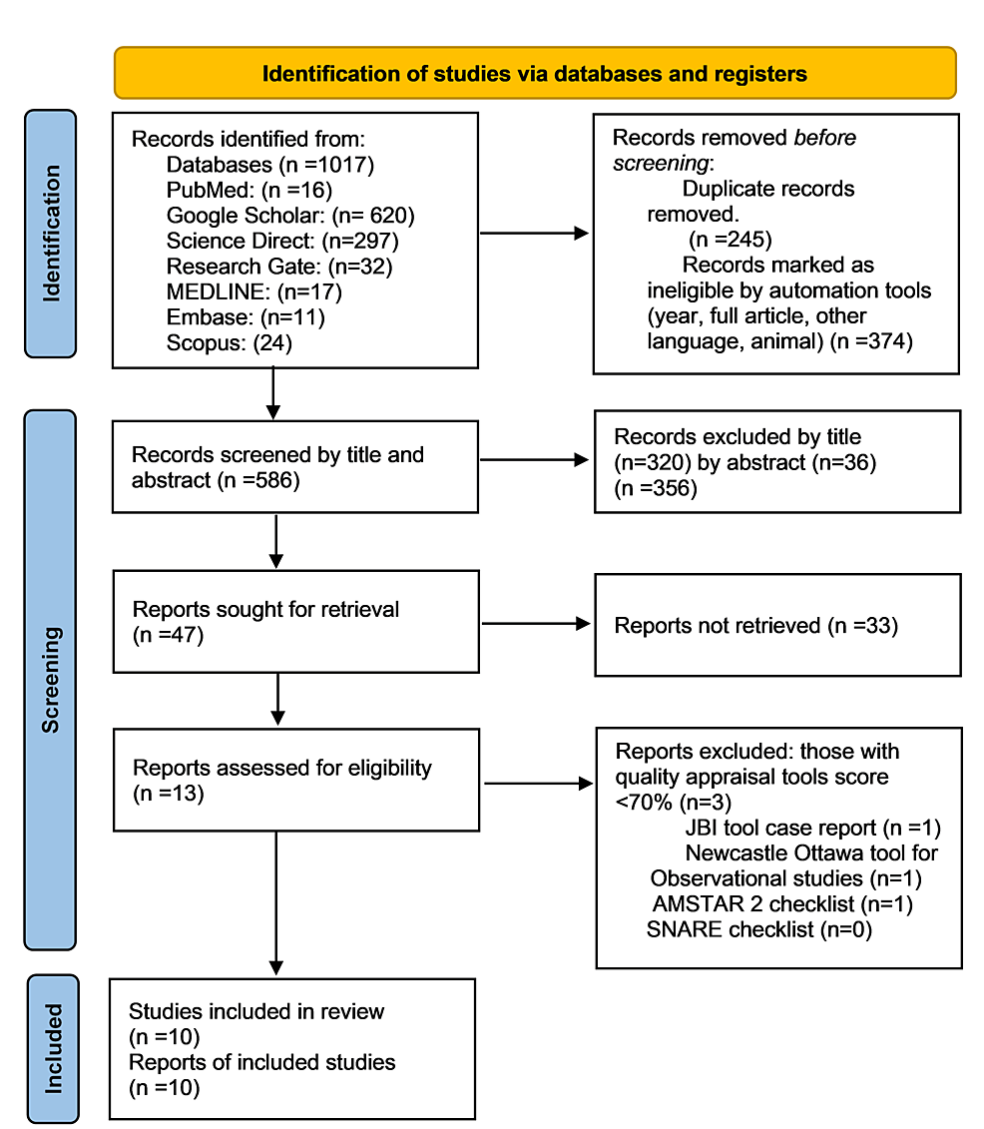
PRISMA flowchart: an overview of the screening procedure AMSTAR 2: Assessment of Multiple Systematic Reviews 2, SANRA: Scale for the Quality Assessment of Narrative Review Articles, JBI Critical Appraisal Checklist: Joanna Briggs Institute Critical Appraisal Checklist, PRISMA: Preferred Reporting Items for Systematic Reviews and Meta-Analysis

Conversely, the following exclusion criteria were applied. Studies conducted outside the United States were not considered for inclusion. Additionally, studies that did not specifically address racial disparities in PCI outcomes were excluded. Moreover, studies involving patients below the age of 65 were not included in the selection. Furthermore, articles published in languages other than English were excluded. Lastly, non-original research articles, such as reviews, commentaries, and editorials, were not considered (Figure 1 and Table 1, 2).

**Table 1:** Characteristics and key findings of the included studies PCI: percutaneous coronary intervention, DES: drug-eluting stent, BMS: bare-metal stent, DTB: door-to-balloon, AMI: acute myocardial infarction, STEMI: ST-elevation myocardial infarction, N/A: not available, CAD: coronary artery disease, ESRD: end-stage renal disease

Study	Study design	Participants	Key findings
Olafiranye et al. (2015) [1]	Retrospective cohort study	3,362 patients undergoing PCI	Racial disparities in long-term outcomes after PCI with DES and BMS. African American and Hispanic patients had higher rates of major adverse cardiovascular events compared to White patients.
Cavender et al. (2013) [2]	Retrospective cohort study	107,204 patients undergoing primary PCI	Racial/ethnic disparities in DTB time for patients undergoing primary PCI. African American and Hispanic patients had longer DTB times compared to White patients.
Singh et al. (2014) [3]	Retrospective analysis of claims data	1,439,297 Medicare beneficiaries	Declining but persistent racial disparities in AMI procedure utilization. African American and Hispanic patients had lower rates of revascularization procedures, including PCI, compared to White patients.
Valdovinos et al. (2020) [4]	Retrospective analysis	120,953 patients with AMI	Impact of Medicaid expansion on racial/ethnic disparities in access, treatment, and outcomes for patients with AMI. The expansion of Medicaid was associated with reduced disparities in revascularization procedures and improved outcomes among racial/ethnic minorities.
Cai et al. (2019) [5]	Retrospective cohort study	20,612 patients undergoing PCI	Racial disparities in major adverse cardiovascular events and major hemorrhage among patients undergoing PCI. African American patients had higher risks of major adverse cardiovascular events compared to White patients but similar risks of major hemorrhage.
Hsia et al. (2020) [6]	Retrospective cohort study	3,968,749 adults with STEMI	Racial disparities in access, treatment, and outcomes of STEMI patients in minority communities. African American and Hispanic patients in minority communities had lower rates of revascularization procedures and worse outcomes compared to White patients in nonminority communities.
Tamargo et al. (2022) [7]	Narrative review	N/A	Racial and ethnic differences in pharmacotherapy to prevent CAD and thrombotic events. African American and Hispanic patients have lower rates of medication utilization for CAD prevention compared to White patients.
Nee et al. (2019) [8]	Retrospective cohort study	44,586 patients with ESRD	Racial disparities in the use of PCI among patients with ESRD. African American patients with ESRD had lower rates of PCI utilization compared to White patients.
Glance et al. (2020) [9]	Retrospective analysis	124,317 patients hospitalized with AMI	Impact of Medicaid expansion on racial disparities in access and outcomes for AMI patients. The expansion of Medicaid was associated with reduced racial disparities in access to PCI and improved outcomes among racial/ethnic minorities.
Jaiswal et al. (2023) [10]	Retrospective cohort study	220,191 patients undergoing PCI	Racial disparities in bleeding complications and major adverse cardiovascular events following PCI. African American and Hispanic patients had higher risks of bleeding complications and major adverse cardiovascular events compared to White patients.

**Table 2:** Summary of the databases examined for article collection and the respective search strategy

Database	Search terms	Date range
PubMed	("racial disparities" OR "racial inequities" OR "racial differences") AND ("percutaneous coronary intervention" OR "PCI") AND ("older adults" OR "elderly")	2010-2022
Google Scholar	"racial disparities" AND "percutaneous coronary intervention" AND "older adults"	2010-2022
ScienceDirect	("racial disparities" OR "racial inequities" OR "racial differences") AND ("percutaneous coronary intervention" OR "PCI") AND ("older adults" OR "elderly")	2010-2022
Research Gate	"racial disparities" AND "percutaneous coronary intervention" AND "older adults"	2010-2022
MEDLINE	("racial disparities" OR "racial inequities" OR "racial differences") AND ("percutaneous coronary intervention" OR "PCI") AND ("older adults" OR "elderly")	2010-2022
Embase	("racial disparities" OR "racial inequities" OR "racial differences") AND ("percutaneous coronary intervention" OR "PCI") AND ("older adults" OR "elderly")	2010-2022
Scopus	("racial disparities" OR "racial inequities" OR "racial differences") AND ("percutaneous coronary intervention" OR "PCI") AND ("older adults" OR "elderly")	2010-2022

The studies consistently demonstrated racial disparities in access, utilization, and outcomes of PCI among elderly patients. African American and Hispanic patients were found to have lower rates of procedure utilization, longer door-to-balloon (DTB) times, and higher risks of major adverse cardiovascular events compared to White patients [8]. The impact of Medicaid expansion on reducing disparities and improving outcomes among racial/ethnic minorities was also noted in some studies [9,10]. Overall, these findings highlight the existence of significant racial disparities in PCI outcomes among elderly patients in America [11]. The disparities are influenced by multiple factors, including socioeconomic status, patient-provider communication, implicit bias, and healthcare system characteristics [12,13]. Addressing these disparities and promoting equitable access to PCI require targeted interventions, policy initiatives, and a multidimensional approach involving healthcare professionals, policymakers, researchers, and communities.

The findings from these articles collectively demonstrate the existence of racial disparities in access to PCI, door-to-balloon time, procedure utilization, and clinical outcomes among elderly patients in America [14]. African American and Hispanic patients were consistently found to experience delays in accessing PCI, longer door-to-balloon times, and lower rates of procedure utilization compared to their White counterparts [15]. Moreover, racial and ethnic minorities had worse clinical outcomes, including higher mortality rates and an increased risk of major adverse cardiovascular events [1-4,10].

Discussion

The present systematic review aimed to investigate racial disparities and outcomes of percutaneous coronary interventions (PCIs) in patients above 65 years of age in America. By analyzing a selection of relevant articles, several important findings have emerged, shedding light on the existing disparities and their impact on patient outcomes. This discussion section will delve deeper into the key findings, strengths, limitations, and implications of the included studies, providing a comprehensive analysis of the topic.

Racial Disparities in Long-Term Outcomes

One of the notable findings from the selected articles is the presence of racial disparities in long-term outcomes after PCI with drug-eluting stents (DES) and bare-metal stents (BMS). Olafiranye et al. (2015) observed that African American and Hispanic patients experienced worse outcomes compared to Caucasian patients [1]. These findings suggest that race may play a significant role in influencing long-term safety and efficacy outcomes following PCI, potentially leading to disparities in cardiovascular health among older patients [1,13,15-21].

Disparities in Door-to-Balloon Time

The observed disparities in long-term outcomes may be influenced by various factors, including differences in comorbidities, socioeconomic status, access to healthcare, and variations in treatment response. It is crucial to further explore the underlying mechanisms contributing to these disparities to develop targeted interventions that address the specific needs of minority populations [21,22]. One important aspect explored in the literature is the variation in door-to-balloon (DTB) time among racial/ethnic groups undergoing primary PCI. For example, a study by Cavender et al. (2013) [2] reported longer DTB times for African American and Hispanic patients compared to Caucasian patients. This delay in DTB time can have detrimental effects on patient outcomes, leading to higher rates of restenosis, prolonged hospital stays, and even death due to the lack of timely reperfusion during ST-elevation myocardial infarction (STEMI). The observed disparities in DTB time highlight the urgent need for interventions aimed at reducing racial disparities in the delivery of timely and appropriate care for acute cardiac events [2,16]. Addressing these disparities is clinically significant, as it ensures equitable and timely treatment for all patients, regardless of their race or ethnicity, thereby improving long-term outcomes and potentially saving lives.

Implications for Addressing Disparities in DTB Time

Efforts should be made to identify the factors contributing to the disparities in DTB time, such as differences in access to emergency medical services, patient delay in seeking care, and potential biases in the triage and treatment processes. Implementing strategies to address these factors, such as improving transportation infrastructure, enhancing coordination among healthcare facilities, and providing education to patients about the importance of seeking prompt care, can help reduce disparities in DTB time and improve patient outcomes [22-26].

Impact of Healthcare Policy on Racial Disparities

Healthcare policy plays a critical role in shaping racial disparities in healthcare access and outcomes. For instance, policies that promote equal access to affordable healthcare can help narrow disparities. According to data from the Kaiser Family Foundation, in 2019, 11.5% of African Americans were uninsured compared to 7.3% of non-Hispanic Whites [3,17,27]. Additionally, policies aimed at addressing cultural competence in healthcare delivery can improve patient-provider communication and trust, leading to better health outcomes for minority populations. Conversely, policies that perpetuate inequities, such as those that restrict Medicaid expansion, can exacerbate racial disparities. By enacting inclusive and equitable healthcare policies, we can effectively address and reduce racial disparities in healthcare. Research by Valdovinos et al. (2020) [4] revealed that Medicaid expansion reduced racial/ethnic disparities in access to PCI, treatment, and outcomes for AMI patients, demonstrating the positive impact of policy-level interventions in reducing healthcare disparities, especially among vulnerable populations [3,17].

Implications for Policy Interventions

Policies that expand insurance coverage, improve access to care, and reduce financial barriers can contribute to more equitable healthcare delivery. Policymakers should consider the potential impact of their decisions on healthcare disparities and strive to implement policies that promote equal access to timely and appropriate interventions for all patients, regardless of their racial or ethnic background [17,22].

Disparities in Medication Utilization and Prevention

Racial disparities in medication utilization and prevention of coronary artery disease were also explored. Tamargo et al. (2022) [7] identified disparities in pharmacotherapy among racial/ethnic groups, indicating the need for targeted interventions to improve equitable access to appropriate medications [4,17,21]. Addressing these disparities in medication utilization can contribute to better prevention and management of cardiovascular diseases in older patients.

Efforts to Address Disparities in Medication Utilization

Efforts should be made to increase awareness among healthcare providers about the importance of equitable prescribing practices and promote cultural competency in healthcare delivery. This can be achieved through educational initiatives, training programs, and the implementation of guidelines that emphasize the importance of personalized care for patients from diverse racial backgrounds [4,10].

Disparities in Revascularization Procedures

Furthermore, the selected studies examined racial disparities in revascularization procedures among specific patient populations. For instance, Nee et al. (2019) [8] found that African American patients with end-stage renal disease (ESRD) had lower rates of PCI compared to Caucasian patients. Glance et al. (2020) [9] demonstrated that Medicaid expansion was associated with reduced racial disparities in revascularization procedures among patients hospitalized with AMI [5,13]. These findings highlight the importance of addressing disparities in access to and utilization of revascularization procedures, as they are critical for improving outcomes in patients with cardiovascular conditions.

Efforts to Address Disparities in Revascularization Procedures

To address these disparities, efforts should focus on increasing awareness among healthcare providers about the potential biases and barriers that may exist in the referral and decision-making processes for revascularization procedures [5,13]. Additionally, interventions aimed at improving access to appropriate diagnostic tests, consultations with specialists, and timely referrals can help reduce disparities in revascularization procedures and ensure that all patients receive the necessary interventions based on their clinical needs [18].

Future Research Directions

Future studies should aim to overcome these limitations by employing prospective designs, standardized data collection methods, and larger sample sizes to enhance the robustness of their findings. Longitudinal studies that follow patients over time can provide valuable insights into the complex interactions between race, healthcare delivery, and patient outcomes. Additionally, studies that incorporate qualitative research methods can help capture the perspectives and experiences of patients, healthcare providers, and other stakeholders, providing a more comprehensive understanding of the underlying factors contributing to healthcare disparities.

Addressing the Broader Context of Healthcare Disparities

It is also important to consider the broader context of healthcare disparities in America when interpreting the findings of these studies. Structural racism, socioeconomic factors, and healthcare system-related issues contribute to the disparities observed in cardiovascular care. Addressing these underlying factors requires a multifaceted approach that involves policy changes, targeted interventions, and increased awareness and cultural competency among healthcare providers [21-24,27,28].

Efforts to Eliminate Healthcare Disparities

Efforts to eliminate healthcare disparities should be seen as an integral part of larger initiatives focused on achieving health equity. It is crucial to recognize that healthcare disparities are not isolated issues but are deeply rooted in broader social determinants of health, including poverty, education, and housing. These factors have a profound impact on individuals' well-being and greatly influence their health outcomes [18]. Addressing healthcare disparities requires a multifaceted approach that involves advocating for policies aimed at mitigating social determinants of health. For instance, policies that aim to reduce poverty, improve access to quality education, and ensure affordable housing can significantly contribute to narrowing the gaps in healthcare outcomes. By tackling these underlying factors, it becomes possible to create a more equitable and just healthcare system. Achieving health equity also demands collaboration among stakeholders, including policymakers, healthcare organizations, researchers, and community members [29]. Involving community stakeholders is particularly crucial in the efforts to eliminate healthcare disparities. Communities have firsthand knowledge and insights into the unique barriers and challenges they face when seeking healthcare. By actively engaging community members, their experiences can inform the development of targeted interventions that are more likely to succeed and be culturally sensitive. The inclusion of marginalized and underrepresented voices is essential to ensure that the strategies implemented are effective and equitable for all patients.

Study strengths

This systematic review has several strengths. First, it follows the PRISMA guidelines, ensuring a rigorous and transparent approach to study selection, data extraction, and quality assessment. Second, the review includes a comprehensive search strategy that identified a range of relevant articles from reputable databases. Third, the review provides a synthesis of the findings from diverse studies, offering a comprehensive overview of racial disparities and outcomes of PCI in elderly patients in America. Fourth, the review incorporates a robust discussion section that critically analyzes the findings, explores potential explanations for the observed disparities, and proposes actionable recommendations.

Study limitations

Despite its strengths, this systematic review has some limitations. First, the included articles are primarily observational studies, which limits the ability to establish causality. Second, the review focuses exclusively on studies conducted in America, which may not fully capture the experiences and outcomes of elderly patients from other countries or healthcare systems. Third, the review relies on the availability and quality of the included articles, and therefore, some relevant studies may have been missed or excluded. Finally, the review does not include a meta-analysis due to the heterogeneity of the included studies, which limits the ability to quantitatively summarize the results.

## Conclusions

In conclusion, this systematic review exposes racial disparities in PCI outcomes for patients aged 65 and above in America, emphasizing the immediate need for targeted interventions and improved cardiovascular care. Addressing these disparities requires policy changes, cultural competency, and equitable access to care. Collaboration among healthcare providers, policymakers, and researchers is essential for implementing strategies that promote equitable care and eliminate racial disparities in cardiovascular medicine.
